# Supplementary material for: The Potential Benefits of Acute Aronia Juice Supplementation on Physical Activity Induced Alterations of the Serum Protein Profiles in Recreational Runners: A Pilot Study
Source: Healthcare (Basel). 2024 Jun 26;12(13):1276. doi: 10.3390/healthcare12131276 (PMC11240927; doi:10.3390/healthcare12131276)
Supplement: Supplementary file 1 [file healthcare-12-01276-s001.zip › healthcare-3066044-supplementary.pdf]

The Potential Benefits of Acute Aronia Juice Supplementation on Physical Activity Induced Alterations of the Serum Protein Profiles in Recreational Runners: A Pilot Study

Tamara Uzelac [1,†], Marija Takić [2,†,*], Vuk Stevanović [2], Nevena Vidović [2], Ana Pantović [2], Petar Jovanović [1,2] and Vesna Jovanović [1,*]

[1] University of Belgrade - Faculty of Chemistry, Department of Biochemistry and Centre of Excellence for Molecular Food Sciences, Studentski trg 12-16, 11000 Belgrade, Serbia; T.U. tamarauzelac31@gmail.com, V.J. vjovanovic@chem.bg.ac.rs

[2] University of Belgrade, Institute for Medical Research, Group for Nutrition and Metabolism, Centre of Research Excellence in Nutrition and Metabolism, National Institute of Republic of Serbia, Tadeuša Košćuškog 1, 11000 Belgrade, Serbia; V.S. vuk.stevanović@imi.bg.ac.rs, N.V. nevenakardum@gmail.com, A.P. jelenkovicana5@gmail.com, P.J petarjovanovichfbu@gmail.com

* Correspondence: author: marijapo2001@gmail.com, marija.takic@imi.bg.ac.rs, tel: +381 113030997

† Equal contribution.

Supplementary material

Content

Figure S1. Native PAAE on a 9% gel of individual serum samples from half-marathon runners

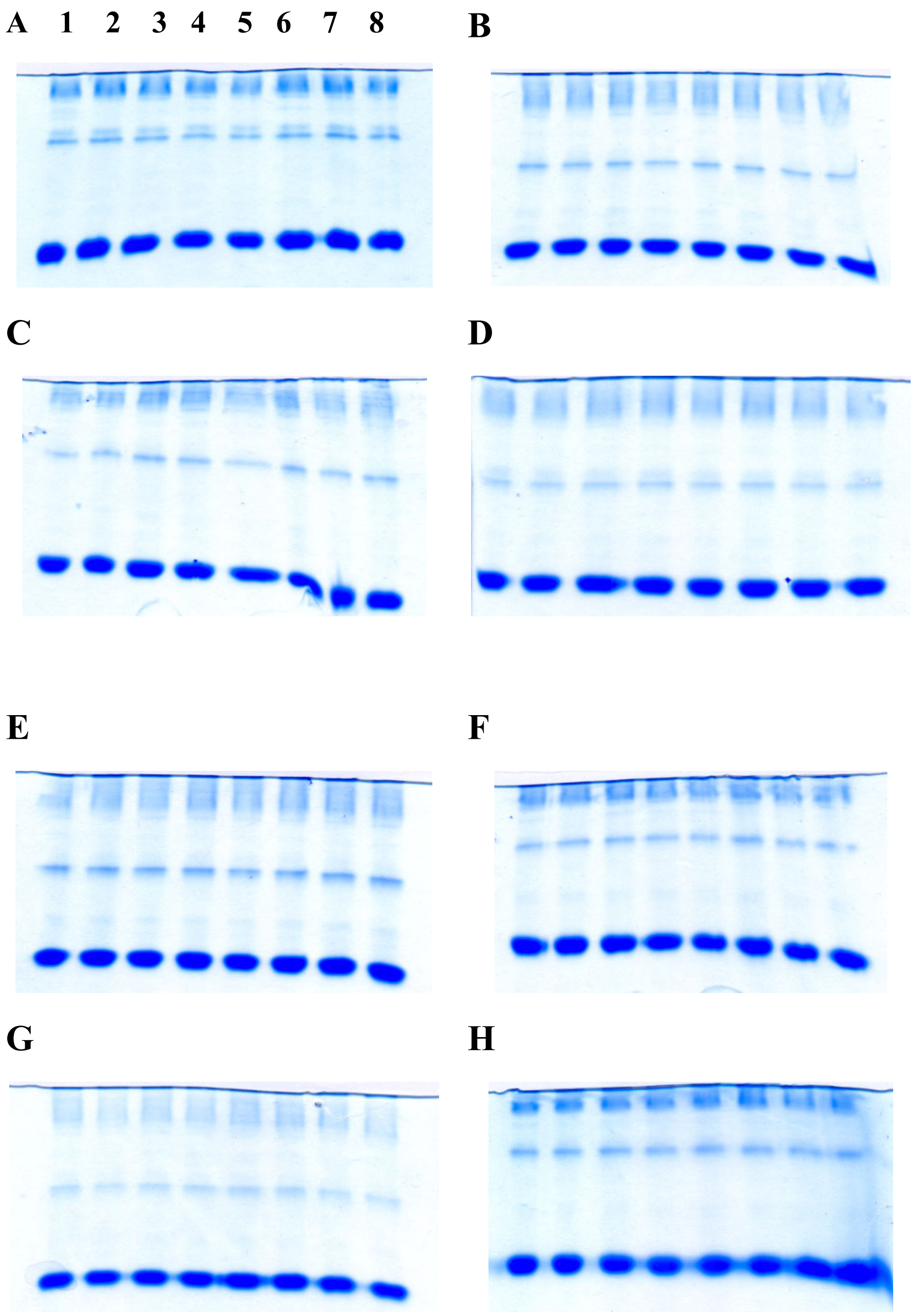

Figure S1. Native PAAE on a 9% gel of individual serum samples from half-marathon runners

(A-J) who did (bars 1-4) and did not (bars 5-8) consume chokeberry juice before (0') and after the race (15 and 60 minutes) and 24 hours. 5 or 7 µg of protein was administered per strip. One plate one runner, the first four lanes
